# MIIP inhibits clear cell renal cell carcinoma proliferation and angiogenesis *via* negative modulation of the HIF-2α-CYR61 axis

**DOI:** 10.20892/j.issn.2095-3941.2020.0296

**Published:** 2021-12-22

**Authors:** Fengqi Yan, Qinhao Wang, Mingyuan Xia, Yi Ru, Wei Hu, Guang Yan, Xin Xiong, Mei Zhang, Jiancai Wang, Qi Li, Bo Zhang, He Wang, Wei Lin, Guojun Wu, Xia Li

**Affiliations:** 1 State Key Laboratory of Cancer Biology, Department of Biochemistry and Molecular Biology, the Fourth Military Medical University, Xi’an 710032, China; 2 Department of Urology, Tang Du Hospital, the Fourth Military Medical University, Xi’an 710038, China; 3 Department of Neurosurgery, Xi Jing Hospital, the Fourth Military Medical University, Xi’an 710032, China; 4 Urology and Nephrology Hospital, Xi’an People’s Hospital, Xi’an 710100, China

**Keywords:** MIIP, ccRCC, HIF-2α, CYR61, HSP90, RACK1

## Abstract

**Objective:**

In various cancers, migration and invasion inhibitory protein (MIIP) is expressed at low level and is involved in cancer pathogenesis. Herein, we sought to explore the function of MIIP in clear cell renal cell carcinoma (ccRCC).

**Methods:**

CCK-8, colony formation, cell cycle, and endothelial cell tube formation assays were performed to evaluate the roles of MIIP in ccRCC proliferation and angiogenesis. To explore the underlying mechanism, we conducted RNA-sequencing, GSEA, qRT-PCR, Western blot, ELISA, cell transfection, coimmunoprecipitation, and ubiquitination assays in ccRCC cell lines. Furthermore, xenograft tumor growth in nude mice, and Ki-67 and CD31 staining in xenograft tissues were examined. Finally, the association of MIIP expression with clinical pathology and the expression status of HIF-2α and cysteine-rich 61 (CYR61) were further analyzed in human RCC tissues through Western blot and immunohistochemistry.

**Results:**

Both *in vitro* and *in vivo* functional experiments indicated that forced expression of MIIP inhibited ccRCC proliferation and angiogenesis, whereas silencing MIIP either in normal HK-2 cells or in ccRCC cells had the opposite effect (*P* < 0.05). Mechanistically, *CYR61* was identified as a gene significantly downregulated by MIIP overexpression, and was required for the suppressive role of MIIP in ccRCC. MIIP was found to promote HSP90 acetylation and thus impair its chaperone function toward HIF-2α. Consequently, RACK1 binds HIF-2α and causes its ubiquitination and proteasomal degradation, thus decreasing the transcription of its target, CYR61. Finally, analyses of clinical samples demonstrated that MIIP is significantly downregulated in cancer *vs*. normal tissues in RCC cases, and its expression is negatively associated with histological grade, metastasis, the prognosis of patients with RCC, and the expression of HIF-2α and CYR61 (*P* < 0.05).

**Conclusions:**

MIIP is a novel tumor suppressor in ccRCC *via* negative regulation of HIF-2α-CYR61 axis.

## Introduction

Renal cell carcinoma (RCC) is among the 10 most common cancers and accounts for approximately 3% of adult malignancies^1^. Worldwide, its incidence has significantly increased over the past 3 decades, at a rate of approximately 2%–3% per decade^2^. Approximately 17% of patients diagnosed with RCC have already reached the metastatic phase^3^. The 5-year survival of metastatic RCC is estimated to be only 5%–15%^4^. Because of resistance to radiotherapy and chemotherapy, antiangiogenic drugs have been used as the primary therapeutic approach for metastatic RCC in recent decades^4^. However, such therapeutics still cannot achieve remission, and resistance to these targeting agents has been frequently reported^5,6^. Therefore, the molecular mechanisms underlying RCC angiogenesis and progression must be further investigated to develop more effective therapies for patients with RCC.

The most common subtype of RCC, accounting for 70%–90% of cases, is clear cell RCC (ccRCC)^7^. ccRCC is a highly vascularized cancer of the kidney, and this vascularization promotes local tumor progression and metastatic spread^8^. This carcinoma is usually associated with inactivation of the von Hippel-Lindau (*VHL*) tumor-suppressor gene, which causes hypoxia inducible factors (HIF) activation^9^. The VHL-encoded protein (pVHL) is a recognition component of the E3 ubiquitin ligase complex, which mediates the ubiquitination and subsequent proteasomal degradation of the α-subunits of HIF^10^. This process also depends on oxygen and prolyl hydroxylase (PHD), which mediates hydroxylation of HIF-1/2α at specific proline residues, thus triggering binding of pVHL^9^. Therefore, hypoxia or VHL inactivation results in the stabilization and accumulation of HIFα isoforms, mainly HIF-1α and/or HIF-2α^10^. HIF-1/2α then translocates into the nucleus and forms a functional heterodimer with the constitutively expressed β subunit (HIF-β), thus leading to the expression of multiple target genes involved in angiogenesis, cell proliferation, and other biological processes^11–13^. HIF-1α and HIF-2α were previously thought to promote tumor progression through largely overlapping functions^10^. However, in recent years, evidence has revealed the unique and sometimes opposing activities of these HIFα isoforms in both normal physiology and disease^9^. Particularly in ccRCC, HIF-1α appears to function as a tumor suppressor, and its expression is often silenced, whereas HIF-2α acts as a driver of ccRCC progression^9,13,14^. However, understanding of the downstream effectors and upstream regulators of HIF-2α is far from complete.

The migration and invasion inhibitory protein (MIIP; also known as IIp45) gene is located on chromosome 1p36.22, which is commonly deleted in a wide spectrum of cancers^15^. MIIP comprises 388 amino acids and contains 3 segments of low compositional complexity domains and an arginine-glycine-aspartate motif^15^. Although its physiological role remains elusive, MIIP is increasingly identified as a novel tumor suppressor. The expression of MIIP is downregulated in many types of cancer. MIIP participates in the regulation of cell migration, invasion, and proliferation, *via* interaction with different partners, such as IGFBP2, Cdc20, histone deacetylase 6 (HDAC6), and PAK1^16–22^. In addition, our recent studies have demonstrated that MIIP is downregulated in prostate cancer, and its overexpression inhibits tumor growth and epithelial-mesenchymal transition by modulating PP1α-AKT signaling and the miR-181a/b-5p-KLF17 axis, respectively^23,24^. These findings strongly suggest that MIIP plays a tumor-suppressive role *via* multifaceted mechanisms in different types of tumors, but the role of MIIP in RCC and its function in tumor angiogenesis remain unclear.

In the present study, we demonstrated that MIIP inhibits the proliferation and proangiogenic capability of ccRCC *in vitro* and *in vivo*. Mechanistically, MIIP facilitates proteasomal degradation of HIF-2α and thus attenuates the HIF-2α-CYR61 axis by promoting HSP90 acetylation and consequently impairing its chaperone function for HIF-2α. Another E3 ubiquitin ligase, RACK1, binds HIF-2α and mediates its ubiquitination and degradation. Analyses of RCC tissue samples showed that MIIP is significantly downregulated in most RCC cases, and its expression was negatively associated with histological grade, metastasis, and the expression of HIF-2α and CYR61. Therefore, MIIP is a novel tumor suppressor in RCC and may serve as a potential therapeutic molecule for RCC treatment.

## Materials and methods

### Cell culture and stable cell lines

The human ccRCC cell lines 786-O and OS-RC-2 and the human kidney proximal tubular cell line HK-2 were purchased from Chinese Academy of Sciences Cell Bank. Human umbilical vascular endothelial cells (HUVECs) were obtained and stored at our department. The 786-O, OS-RC-2, and HK-2 cells were maintained in RPMI-1640 medium (Gibco, USA). HUVECs were grown in ECM (ScienCell, USA). All media were supplemented with 10% fetal bovine serum (Gibco), penicillin, and streptomycin. All cells were incubated in a 37 °C humidified incubator with 5% CO_2_.

MIIP-overexpressing OS-RC-2 and 786-O cell lines (OS-RC-2-MIIP and 786-O-MIIP) and MIIP-knockdown HK-2 and 786-O cell lines (HK-2-shMIIP #1, HK-2-shMIIP #2; 786-O-shMIIP #1 and 786-O-shMIIP #2) were generated by infecting cells with a lentivirus for expression of MIIP (pLEX-MIIP-HA) or MIIP-specific short hairpin RNA (shRNA) (pLKO.1-shMIIP #1 and pLKO.1-shMIIP #2), which were constructed as described in the **Supplementary methods**. The control cells (OS-RC-2-Vector, 786-O-Vector; HK-2-Scrambled and 786-O-Scrambled) were generated by infecting cells with the corresponding control lentivirus (pLEX-HA and pLKO.1-Scrambled). At 48 h after lentivirus infection, the cells were selected with puromycin (Santa Cruz Biotechnology, USA) for 3 weeks. The primers used for plasmid construction are listed in **Supplementary Table S1**.

### Plasmid or siRNA transfection

Chemically synthesized siRNA (si-CYR61, si-HIF-2α, si-RACK1, and si-NC) duplexes were purchased from Gene Pharma (Shanghai, China). The plasmids (pcDNA3.1-HA-CYR61 and pcDNA3.1-Flag-HIF2A) were obtained from Hanbio (Shanghai, China). pCMV-MIIP and pcDNA3.1(+)-3×HA-Ub were the kind gifts from Dr. Wei Zhang (Wake Forest Baptist Medical Center, Winston-Salem, NC, USA) and Dr. David Dornan (Genentech, Inc., South San Francisco, CA, USA), respectively. Transfection of siRNAs and plasmids with Lipofectamine 2000 (Thermo Scientific, Waltham, MA, USA) was conducted according to the manufacturer’s protocol. The siRNA sequences are listed in **Supplementary Table S2**.

### Western blot

Cell lysates were obtained through lysis of cultured cells or tumor samples with RIPA buffer (Beyotime, China; protease inhibitor cocktail, Roche, Switzerland). The secreted proteins were harvested by centrifugation of cell culture supernatants as described in the **Supplementary methods**. The concentration of proteins was determined with the BCA method (Beyotime). Equal amounts of protein (10–40 µg) were separated on 8%–15% SDS-PAGE gels (100 V for 90–120 min) and then electro-transferred (300 mA for 90–120 min) to nitrocellulose filter membranes. After blocking with 5% nonfat milk for 1.5 h, the membranes were probed with primary antibodies [anti-MIIP, 1:1000, Sigma, USA; anti-GAPDH, 1:1000, Proteintech, China; anti-CYR61, 1:1000, Proteintech add the nation; and anti-HIF-2α, 1:1000, Proteintech/Cell Signaling Technology (CST) add the nation] at 4 °C for 12 h. All nitrocellulose membranes were then incubated with goat-anti-rabbit or goat-anti-mouse horseradish peroxidase-conjugated secondary antibodies for 1.5 h at room temperature. The signals of the protein bands were detected with the ECL Imaging System (Tanon, China).

### Cell proliferation, colony formation, and cell cycle analyses

Cells were seeded into 96-well plates in septuplicate at 1 × 10^3^ per well, and cell viability was tested with a CCK-8 Kit (Dojindo, Japan) every 24 h. The absorbance of each well was measured at 450 nm with a microplate reader (Bio-Rad, USA). Cell colony formation was measured 2 weeks after cells were seeded into 6 cm dishes at 5 × 10^2^ per dish. Cell colonies were fixed with 4% paraformaldehyde solution and stained with crystal violet dye. The colony number was calculated. For cell cycle analysis, cells were cultured with serum-free RPMI-1640 medium for 24 h to synchronize the cell cycle. Then the serum-free RPMI-1640 medium was replaced with RPMI-1640 medium containing 10% serum, and the cells were cultured for 48 h. Finally, the cells were fixed in 75% ethanol, stained with propidium iodide, and analyzed with a flow cytometry system.

### Endothelial cell tube formation

HK-2 or ccRCC cells (2 × 10^5^) were cultured on 6-well plates with 2 mL fresh RPMI-1640 medium. After 24 h, the medium was collected and centrifuged at 1,000 r/min for 5 min to remove any cell debris before its use as conditioned medium (CM). The 48-well plates were precoated with Matrigel (BD USA) and kept at 37 °C for 30 min. Then 8 × 10^4^ HUVECs were suspended in 200 µL CM, then seeded on collagen-coated 48-well plates for 5 h. Finally, images were taken under a microscope (Olympus Japan), and the number of capillary-like tubes was counted in Image J software.

### RNA extraction, RNA-sequencing, and real-time PCR (qRT-PCR)

Total RNA was extracted from cells with TRIzol reagent (Invitrogen, USA). RNA-seq was performed with 786-O-Vector and 786-O-MIIP cells, and the data were analyzed by Beijing Novogene Biological Information Technology Company (Novogene, Beijing, China). For qRT-PCR, cDNA was synthesized from total RNA (1 µg) with a PrimeScript™ RT Reagent Kit [TaKaRa Biotechnology (Dalian), China]. qRT-PCR was conducted with a Real-Time Detection System (Bio-Rad) with SYBR Green II (TaKaRa). The conditions were as follows: 95 °C for 1 min, followed by 40 cycles of 95 °C for 10 s and 60 °C for 30 s. Raw data were normalized to the internal *GAPDH* levels and presented as the relative expression level of the gene of interest, as calculated by the 2^-ΔΔCt^ method. The primers are listed in **Supplementary Table S3**.

### ELISA

The concentration of the CYR61 protein released in the supernatant was tested with a commercial CYR61 ELISA kit (Boster, China). Cells were seeded into 6-well plates at 2 × 10^5^ per well and cultured in 2 mL medium for 24 h. Then the culture supernatants were harvested and transferred to a 96-well ELISA plate (100 µL per well) and incubated at 37 °C for 90 min. Afterward, the supernatants were aspirated, and the plate was incubated with anti-CYR61 for another 60 min at 37 °C, then incubated with ABC solution within 30 min. The TMB solution was added to the well, and the plate was incubated in the dark for 20 min. The optimal density reading was determined with a microplate reader at 450 nm.

### Coimmunoprecipitation and ubiquitination assays

For the coimmunoprecipitation assays, lysates of 786-O-Vector and 786-O-MIIP cells were incubated with anti-HIF-2α or anti-HSP90 antibodies, followed by protein A + G Sepharose beads. The precipitates were resolved with SDS-PAGE and subjected to Western blot with anti-HIF-2α (1:1000, Proteintech/CST), anti-HSP90 (1:1000, Proteintech), anti-RACK1 (1:1000, Proteintech), anti-MIIP, or anti-acetyl lysine (1:1000, Abcam). For the ubiquitination assays, 786-O cells (1 × 10^6^) were seeded onto 10 cm dishes with 10 mL fresh RPMI-1640 medium and cultured in an incubator. After 24 h, 786-O cells were transiently cotransfected with 5 µg pcDNA3.1(+)-3×HA-Ub and 10 µg pCMV-MIIP or pCMV; 48 h later, the cells were treated with 10 µM MG132 for 4 h before harvesting. Cell lysates were then immunoprecipitated with anti-HIF-2α antibody as described above and subjected to Western blot analysis with anti-HA (1:1000, Cell Signaling Technology) and anti-HIF-2α (1:1000) antibodies.

### Subcutaneous cell implantation in nude mice

The animal studies were approved by the Animal Ethics Committee of the Fourth Military Medical University (Approval No. 20180315). Six-week-old male BALB/c nude mice (five mice per group) were bilaterally subcutaneously injected in the groin with 1 × 10^7^ ccRCC cells (OS-RC-2-MIIP *vs.* OS-RC-2-Vector; 786-O-MIIP *vs.* 786-O-Vector; 786-O-shMIIP 1# *vs.* 786-O-Scrambled), which were resuspended in 200 µL 50% Matrigel medium (BD USA). Tumor growth was monitored every 3 days, and tumor volume was calculated with a standard formula: tumor volume (mm^3^) = width^2^ (mm^2^) × length (mm) × 0.5. Thirty days later, the mice were sacrificed, and their subcutaneous tumors were harvested, measured, photographed, and fixed for further histopathological analyses.

### Patient tissue samples

All samples were collected with the informed consent of the patients, and the experiments were approved by the Research Ethics Committee (Approval No. KY20180403-1), Xijing Hospital, the Fourth Military Medical University (Shaanxi, Xi’an, China). Tissue microarrays containing normal renal and RCC paraffin tissue samples (50 samples for normal, 157 samples for carcinoma) were obtained from the Department of Pathology of Xijing Hospital (Shaanxi, Xi’an, China). Fresh tumor tissues with adjacent normal renal tissues from 13 patients with RCC, used for Western blot analysis, were obtained from the Department of Urology of Xijing Hospital (Shaanxi, Xi’an, China). All samples were subjected to histologic evaluation by pathologists and diagnosed according to the World Health Organization classification (WHO Fourth Edition published in 2016).

### Immunohistochemistry and immunofluorescence staining

Immunohistochemistry was performed on human RCC tissues and xenografted tumor tissues with anti-MIIP (1:100), anti-CYR61 (1:200), anti-HIF-2α (1:200), or anti-Ki67 (1:200, Proteintech) primary antibodies. Immunofluorescence staining was performed on xenografted tumor tissues with anti-CD31 (1:200, Proteintech) primary antibodies. The staining score was calculated by multiplying the stained area (%) score and the intensity score. The stained area (%) score was based on the percentage of cells with positive staining (<5%: 0; 5%–25%: 1; 26%–50%: 2; 51%–75%: 3; and >75%: 4), and the intensity score was based on the cell staining intensity (no staining: 0; weak: 1; moderate: 2; and strong: 3). High expression was specified by a score ≥9, whereas low expression corresponded to a score <9. All scoring work was performed independently by 2 pathologists.

### Statistical analysis

The results were analyzed in SPSS 19.0 software and GraphPad Prism 6 software. All experiments were performed at least 3 times. Quantitative data are expressed as the mean ± standard deviation (SD). Independent Student’s *t*-test or *χ*^2^ test was used to compare the data between 2 groups or more than 2 groups. *P < 0.05 and **P < 0.01 were considered to indicate statistically significant differences.

## Results

### MIIP inhibits the proliferation and proangiogenic activity of ccRCC cells *in vitro*

**Figure 1:**
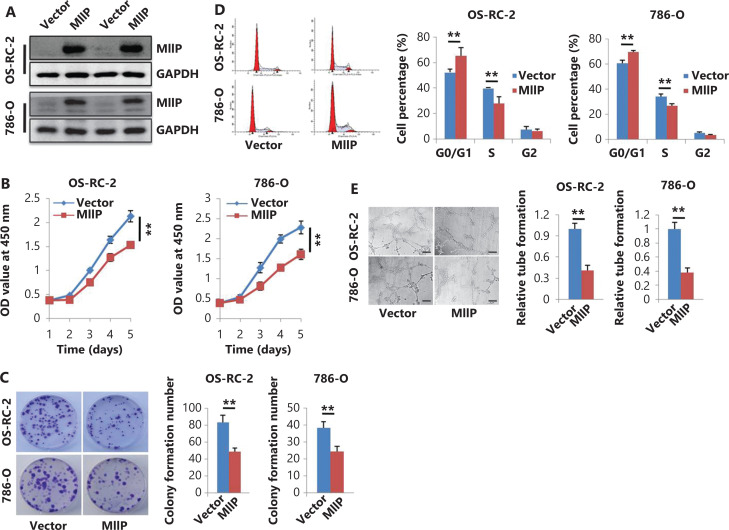
MIIP overexpression inhibits the proliferation and proangiogenic activities of ccRCC cells *in vitro*. Cells stably overexpressing MIIP (OS-RC-2-MIIP or 786-O-MIIP) and control cells (OS-RC-2-Vector or 786-O-Vector) were generated by lentiviral infection. (A) Stable MIIP expression in cells was detected with Western blot. (B) Viability was measured with CCK-8. (C) Colony forming ability was detected with colony formation assays. (D) Cell cycle profiles were analyzed by flow cytometry. (E) Proangiogenic activity was measured with HUVEC tube formation assays, and the HUVECs were cocultured with conditioned medium for 5 h (scale bar: 100 μm). In (C)–(E), the data are represented as mean ± SD, *n* = 3. **P < 0.01.

To determine the role of MIIP in ccRCC cells, we first established MIIP-stably overexpressing OS-RC-2 and 786-O and MIIP-silenced HK-2 and 786-O cell lines through lentivirus infection, on the basis of the endogenous expression of MIIP in HK-2 cells and several ccRCC cell lines (**Supplementary Figure S1A**). The expression of MIIP in these cell lines was confirmed by Western blot (**Figure 1A and Supplementary Figure S1B**). Then the proliferation of ccRCC cells and HK-2 cells was detected. As shown in **Figure 1B**, CCK-8 assays showed that MIIP overexpression significantly inhibited the proliferation of OS-RC-2 and 786-O cells (*P* < 0.01). In contrast, knockdown of MIIP promoted the proliferation of normal HK-2 cells as well as ccRCC 786-O cells (*P* < 0.01) (**Supplementary Figure S1C**). These results were further confirmed by colony formation assays and flow cytometry analysis, which showed that the OS-RC-2-MIIP and 786-O-MIIP cells had significantly lower colony formation capability (*P* < 0.01) (**Figure 1C**) and a higher proportion of G1/G0 phase cells (*P* < 0.01) (**Figure 1D**) than the corresponding control cells. In contrast, MIIP silencing in HK-2 and 786-O cells had the opposite effects on colony formation and cell cycle distribution (*P* < 0.01) (**Supplementary Figure S1D–E**). These results indicated that MIIP functions as an inhibitor of cell proliferation.

Because angiogenesis is a key feature of RCC^25^, we wondered whether MIIP might influence the proangiogenic activity of ccRCC cells. To this end, we suspended HUVECs in CM from cells with stable MIIP overexpression or silencing, and evaluated the complete tubular structures formed by HUVECs. As shown in **Figure 1E**, CM from MIIP-overexpressing 786-O and OS-RC-2 cells, compared with control cells, showed a significantly lower average number of complete tubular structures formed by HUVEC, whereas CM from MIIP-silenced HK-2 and 786-O cells had the opposite effect (*P* < 0.01) (**Supplementary Figure S1F**). These results indicated that MIIP negatively affects the proangiogenic activity of ccRCC cells and HK-2 cells. Overall, MIIP plays a key role in inhibiting the proliferation and angiogenesis of ccRCC cells.

### MIIP negatively regulates CYR61 expression

**Figure 2:**
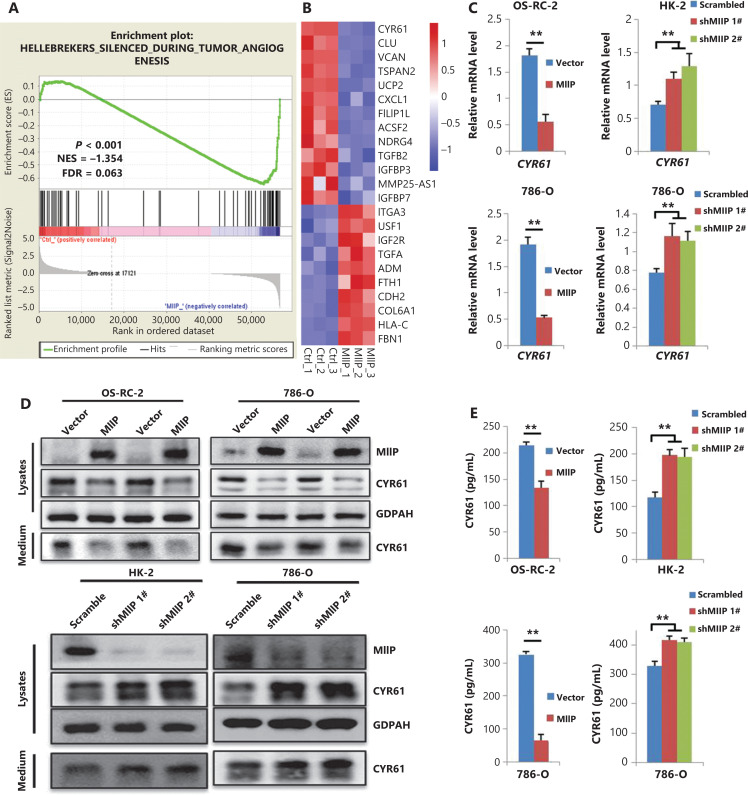
MIIP negatively regulates CYR61 expression. (A) RNA-seq analyses were performed in triplicate on 786-O-MIIP and 786-O-Vector cells, and the datasets were analyzed by GSEA. Tumor angiogenesis pathway GSEA (hallmark gene sets) of the RNA-seq dataset is shown. (B) Heatmap of expression changes in a portion of angiogenesis genes determined from GSEA analysis. (C) mRNA levels of CYR61 in MIIP-overexpression and MIIP-knockdown cells were detected with qRT-PCR. (D) Protein levels of MIIP and CYR61 in lysates (Lys) or culture medium (Medium) were detected by Western blot. (E) Protein levels of CYR61 secreted in the culture medium were determined by ELISA. In (C) and (E), the data are represented as mean ± SD, *n* = 3. **P < 0.01.

To explore the underlying mechanism through which MIIP exerts its inhibitory effect on ccRCC cells, we performed RNA sequencing (RNA-Seq) in 786-O-MIIP *vs.* 786-O-Vector cells, given that the 786-O cell line has been widely used as a model of representative VHL-mutant ccRCC cells^26,27^. In agreement with the *in vitro* experiments, gene set enrichment analysis (GSEA) showed that the tumor angiogenesis and hypoxia gene sets were negatively correlated with MIIP expression (**Figure 2A and Supplementary Figure S2A**). Furthermore, we analyzed the differentially expressed genes between 786-O-MIIP and 786-O-Vector cells in the RNA-Seq results. A total of 2,244 genes showed changed expression (fold change ≥2.0), of which 1,225 were upregulated and 1,019 were downregulated by MIIP (**Supplementary Figure S2B**). Among them, cysteine-rich 61 (CYR61, also named CCN1) attracted our attention because it was located in tumor angiogenesis signaling pathway gene sets and was significantly downregulated by MIIP overexpression (**Figure 2B and Supplementary Figure S2B**). To validate the CYR61 downregulation by MIIP, we performed qRT-PCR in ccRCC cells and HK-2 cells with stable overexpression or silencing of MIIP. Accordingly, we found that the expression of CYR61 mRNA was downregulated in MIIP-overexpressing 786-O and OS-RC-2 cells, but upregulated in MIIP-silenced HK-2 and 786-O CYR61 cells (*P* < 0.01) (**Figure 2C**). We further examined the CYR61 protein levels in cell lysates and cell culture medium by Western blot (**Figure 2D**) and ELISA (*P* < 0.01) (**Figure 2E**) and obtained similar results. Together, these data suggested that MIIP negatively regulates the expression of CYR61 in HK-2 and ccRCC cells.

### MIIP exerts its inhibitory effects through inhibiting CYR61

**Figure 3:**
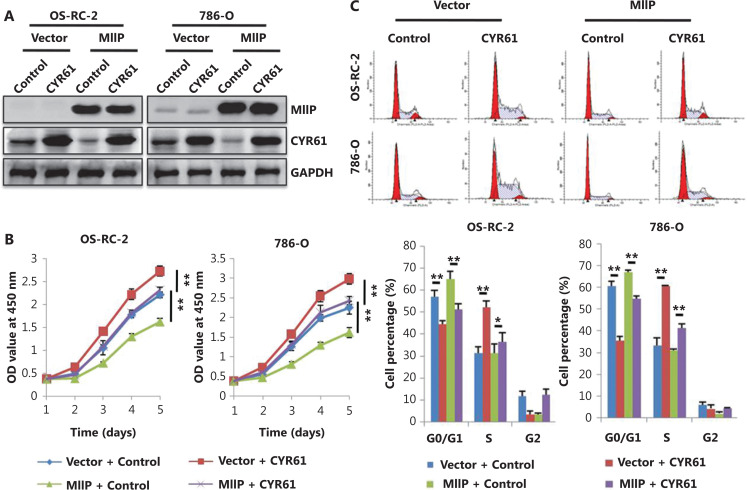

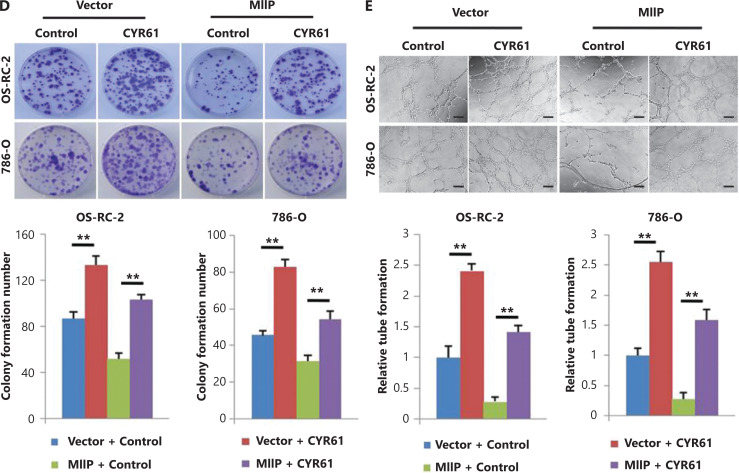
Restoration of CYR61 reverses the inhibitory role of MIIP in ccRCC cells. Cells stably overexpressing MIIP OS-RC-2 or 786-O, or their control cells were transfected with pCDNA3.1-HA-CYR61 or pCDNA3.1, respectively. (A) Protein levels of MIIP and CYR61 were detected by Western blot. (B) Viability was measured with CCK-8. (C) Cell cycle profiles were analyzed by flow cytometry. (D) Colony formation ability was detected with colony formation assays. (E) Proangiogenic activity was measured with HUVEC tube formation assays, and the HUVECs were cocultured with conditioned medium for 5 h (scale bar: 100 μm). In (B)–(E), the data are represented as mean ± SD, *n* = 3. *P < 0.05; **P < 0.01.

Next, we determined whether MIIP inhibits the proliferation and proangiogenic activity of ccRCC by inhibiting CYR61. For this purpose, we forced the expression of CYR61 in MIIP-overexpressing/Vector control ccRCC cells (**Figure 3A**) or silenced CYR61 in MIIP-knockdown/Scrambled control cells (**Supplementary Figure S3A**) to observe the effects on cell proliferation and proangiogenic activities. CYR61 overexpression in both 786-O and OS-RC-2 control cells significantly promoted cell proliferation, colony formation, and proangiogenic activities, whereas MIIP overexpression had opposite effects (*P* < 0.05). Moreover, restoring CYR61 expression in MIIP-overexpressing cells abrogated the inhibitory effect of MIIP on these cellular behaviors (*P* < 0.05) (**Figure 3B–E**). In contrast, CYR61 silencing inhibited cell proliferation and the proangiogenic ability of HK-2 and 786-O cells; similarly, CYR61 silencing attenuated MIIP knockdown-induced alterations in these cellular behaviors (*P* < 0.01) (**Supplementary Figure S3B–E**). Thus, we concluded that CYR61 promotes ccRCC cell proliferation and proangiogenic activity and is a key mediator of the function of MIIP in ccRCC cells.

### MIIP enhances HIF-2α degradation and attenuates the HIF-2α-CYR61 axis in ccRCC cells

**Figure 4:**
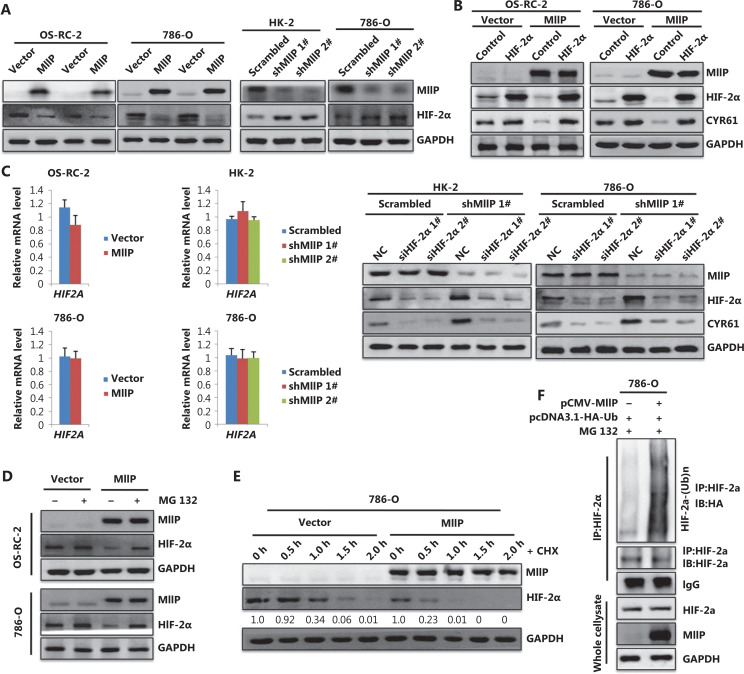
MIIP down-regulates the expression of CYR61 by enhancing the degradation of HIF-2α (A) The protein levels of HIF-2α in MIIP-overexpression and MIIP-knockdown cells were detected by Western blot. (B) Cells stably overexpressing MIIP and control cells were transfected with pCDNA3.1-Flag-HIF2A (HIF-2α) or pCDNA3.1 (control), respectively; cells with stable MIIP knockdown and control cells were transfected with siRNA targeting HIF-2α (siHIF-2α 1# or siHIF-2α 2#) or control (siNC), respectively. HIF-2α and CYR61 protein levels were detected by Western blot. (C) mRNA levels of *HIF2A* in MIIP-overexpressing and MIIP-knockdown cells were detected by qRT-PCR. Data represent mean ± SD, *n* = 3. (D) MIIP-overexpressing OS-RC-2 and 786-O cells, and control cells were treated with MG132 (10 μM) or mock (DMSO). MIIP and HIF-2α levels were detected with Western blot. (E) MIIP-overexpressing 786-O cells and control cells were treated with 100 μg/mL CHX at the indicated time points. MIIP and HIF-2α levels were detected with Western blot. (F) The 786-O cells were co-transfected with plasmids expressing HA-Ub and MIIP and treated with MG-132. HIF-2α ubiquitination was assessed with *in vivo* ubiquitination assays, *via* immunoprecipitation with anti-HIF-2α antibody followed by immunoblotting with anti-HA antibody.

CYR61 has been reported to be transcriptionally regulated by HIF proteins and to mediate the proangiogenic activity of VHL-mutant renal carcinoma cells^28,29^. Here, GSEA indicated a negative correlation between MIIP expression and the hypoxia signaling pathway in ccRCC (**Supplementary Figure S2A**). Of note, 786-O cells lack HIF-1α, whereas both OS-RC-2 and 786-O cells exhibit high levels of HIF-2α^30^, the predominant isoform in ccRCC^9,13,14^. These findings prompted us to ask whether MIIP might downregulate the expression of CYR61 *via* HIF-2α. To test this possibility, we examined HIF-2α protein levels in MIIP-manipulated stable cell lines and found that HIF-2α was significantly decreased by MIIP overexpression, but was increased by MIIP knockdown, in ccRCC cells and HK-2 cells (**Figure 4A**). Furthermore, the downregulation of CYR61 in both OS-RC-2-MIIP and 786-O-MIIP stably expressing cells was reversed by forced expression of HIF-2α (**Figure 4B**). In contrast, the increase in CYR61 in MIIP-silenced HK-2 and 786-O cells was blocked by HIF-2α knockdown (**Figure 4B**). In addition, in agreement with findings from a previous study^28^, we confirmed that CYR61 is a target gene of HIF-2α in ccRCC cells, because overexpressing or silencing the expression of HIF-2α can correspondingly increase or decrease the expression level of CYR61(**Figure 4B**). Collectively, these results indicated that MIIP inhibited CYR61 expression *via* HIF-2α.

To address whether MIIP inhibits HIF-2α at the transcriptional or posttranscriptional level, we performed qRT-PCR to detect *HIF2A* mRNA levels. As shown in **Figure 4C**, neither overexpression nor silencing of MIIP affected the mRNA level of *HIF2A* in ccRCC and HK-2 cells, thus indicating that MIIP regulates HIF-2α at the posttranscriptional level. Then we examined whether MIIP promoted HIF-2α degradation. To this end, MIIP-overexpressing OS-RC-2 and 786-O cells and their control cells were treated with the proteasome inhibitor MG132 or mock (DMSO) and then subjected to Western blot analysis of HIF-2α. As we speculated, the decrease in HIF-2α protein after MIIP overexpression was substantially reversed by the treatment of proteasome inhibitor MG132 (**Figure 4D**). These results suggested that MIIP promotes a decrease in HIF-2α in a proteasome-dependent manner. Next, MIIP-overexpressing and control 786-O cells were treated with CHX, a protein synthesis inhibitor, and the stability of HIF-2α in these cells was compared. The results showed that HIF-2α was more unstable in 786-O-MIIP than in control cells (**Figure 4E**), thus implying that MIIP promotes HIF-2α degradation. Because ubiquitination is a prerequisite for protein degradation *via* proteasomes, we next determined whether MIIP might facilitate HIF-2α ubiquitination in *in vivo* ubiquitination assays. The results showed that HIF-2α polyubiquitination, as evidenced by a smear, was clearly greater in MIIP-overexpressing cells than control cells (**Figure 4F**). Collectively, these data indicated that MIIP downregulates HIF-2α protein by enhancing HIF-2α ubiquitination and subsequent proteasomal degradation.

### MIIP facilitates HIF-2α binding to RACK1 rather than HSP90, and promotes HSP90 acetylation

**Figure 5:**
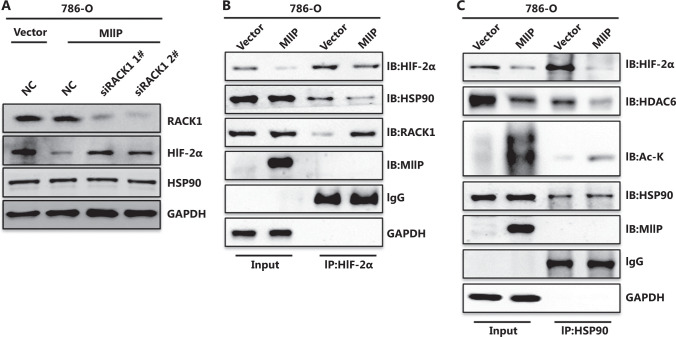
MIIP facilitates HIF-2α binding to RACK1 rather than HSP90, and promotes HSP90 acetylation. (A) Cells stably overexpressing MIIP 786-O and control cells were transfected with siRACK1 1#, siRACK1 2#, or control (siNC) siRNA. The expression of RACK1, HIF-2α, and HSP90 was detected by Western blot. (B) HIF-2α binding to RACK1 and HSP90 was detected with co-immunoprecipitation assays. The 786-O-MIIP and 786-O-Vector cell lysates were immunoprecipitated with anti-HIF-2α antibody, then immunoblotted for RACK1 and HSP90. (C) The acetylation of HSP90, and HSP90 binding to HDAC6 and HIF-2α in 786-O-MIIP and 786-O-Vector cells was detected by immunoprecipitation with anti-HSP90 antibody followed by immunoblotting with anti-Acetyl Lysine, anti-HDAC6, and anti-HIF-2α antibodies.

In addition to pVHL^31^, receptor for activated C-kinase 1 (RACK1) is a component of the E3 complex that mediates proteasomal degradation of HIF-1/2α^32^. Because the ccRCC cell lines that we used were pVHL deficient, we asked whether RACK1 might be involved in this process. We knocked down RACK1 in 786-O-MIIP cells and subsequently observed blocked HIF-2α downregulation (**Figure 5A**), thus suggesting that the MIIP-enhanced HIF-2α degradation relies on RACK1. Because RACK1 facilitates proteasomal degradation of HIF-1/2α by competing with HSP90 for binding^32^, we sought to determine whether MIIP might affect this competitive binding to HIF-2α by performing coimmunoprecipitation assays. The results clearly showed that HIF-2α interacted more with RACK-1 but less with HSP90 in 786-O-MIIP than in 786-O-Vector cells, whereas there was no difference in RACK-1 and HSP90 protein levels between these cells, and no interaction between HIF-2α and MIIP (**Figure 5B**). These findings suggested that MIIP may disrupt the chaperone function of HSP90 toward HIF-2α. Acetylation of HSP90, a reversible modification mediated by opposing actions of acetyltransferases and deacetylases, disrupts the role of this protein as a molecular chaperone in protein maturation and stability^33–35^. Given that HSP90 is a known substrate of HDAC6, and that MIIP inhibits the stability and deacetylase activity of HDAC6^19^, we then asked whether MIIP might affect HSP90 acetylation status *via* HDAC6. The results of the coimmunoprecipitation assays showed that in MIIP-overexpressing cells, the HSP90 acetylation signal was markedly increased and was accompanied by decreased binding of HSP90 to HIF-2α and HDAC6 (**Figure 5C**). Collectively, these data indicated that MIIP promotes HSP90 acetylation by inhibiting HDAC6 activity, thus impairing the chaperone function of HSP90 and its binding to HIF-2α, and consequently increasing RACK1 binding and subsequent HIF-2α degradation.

### MIIP suppresses tumor growth and angiogenesis of ccRCC *in vivo*

**Figure 6:**
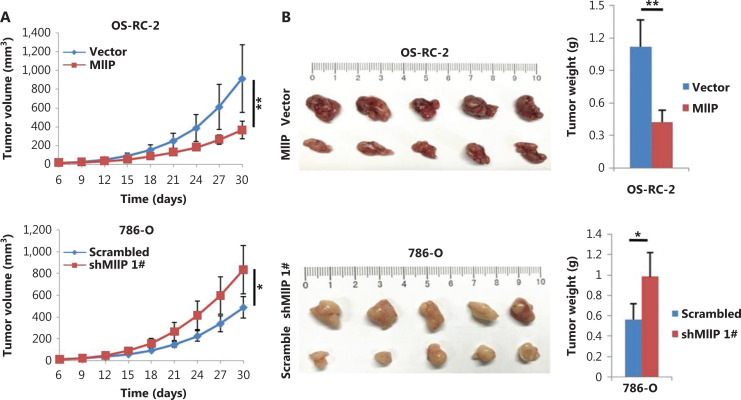

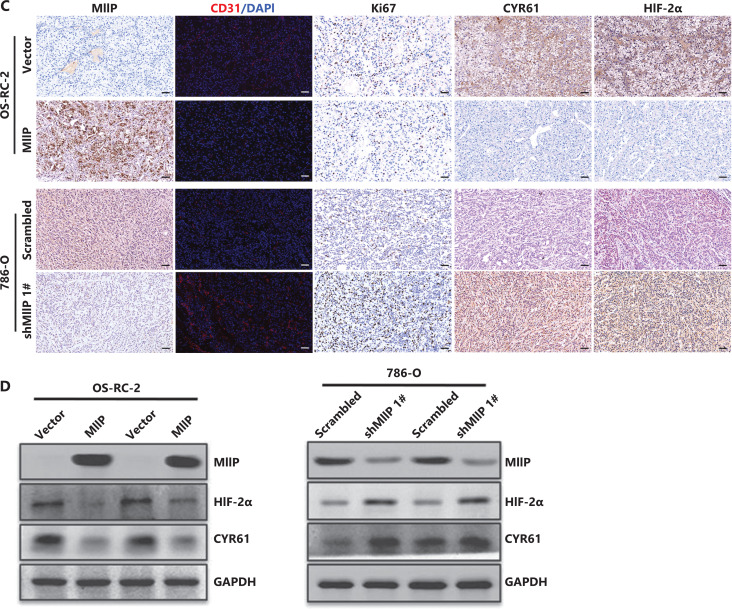
MIIP suppresses tumor growth and angiogenesis of ccRCC *in vivo.* OS-RC-2-MIIP, 786-O-shMIIP 1#, and their corresponding control cells were subcutaneously bilaterally injected into the groin in nude mice (five mice per group). (A) Tumor growth was monitored by caculation of tumor volumes with the formula width^2^ × length × 0.5 along time. (B) The tumor xenografts were photographed, and their weights were measured at the end of the experiment. (C) MIIP, CD31, Ki67, HIF-2α, and CYR61 levels in xenograft tissues were examined by immunohistochemistry or immunofluorescence staining (scale bar: 50 μm). (D) MIIP, HIF-2α, and CYR61 levels in xenograft tissues were detected by Western blot. In (A, B), the data are represented as mean ± SD, *n* = 5. *P < 0.05; **P < 0.01.

To further confirm the roles of MIIP in ccRCC *in vivo*, we subcutaneously bilaterally injected OS-RC-2-MIIP, 786-O-MIIP, 786-O-shMIIP #1, and their corresponding control cells into the groin in nude mice to assess tumor development. In agreement with the *in vitro* results, the tumors of the mice injected with MIIP-overexpressing OS-RC-2 and 786-O cells grew more slowly than those of the control groups (*P* < 0.01) (**Figure 6A and Supplementary Figure S4A**). In contrast, the tumors of the mice injected with MIIP-silenced 786-O cells grew faster than those of the control groups (*P* < 0.05) (**Figure 6A**). Consequently, the final tumor size in the MIIP-overexpression groups was significantly smaller than that in the control groups, meanwhile tumor size in the MIIP-silenced groups was larger than that in the scramble groups (*P* < 0.05) (**Figure 6B and Supplementary Figure S4B**). Moreover, Ki67 staining analysis of xenograft tumor tissues revealed significantly fewer Ki67-positive cells in the MIIP-overexpressing groups than in the control groups, whereas silencing MIIP in 786-O cells had the opposite effect (**Figure 6C and Supplementary Figure S4C**). These data suggested that MIIP inhibits ccRCC cell proliferation *in vivo*. Furthermore, immunofluorescence staining showed that CD31, an endothelial marker indicative of tumor angiogenesis, exhibited significantly lower intensity in xenograft tumors formed by OS-RC-2-MIIP and 786-O-MIIP cells than in the control cells (**Figure 6C and Supplementary Figure S4C**). The CD31 signal in xenograft tumor tissues from 786-O-shMIIP #1 cells was much stronger than that from the control cells (**Figure 6C**). These findings provided further support that MIIP inhibits ccRCC angiogenesis *in vivo*.

Next, we verified the expression of HIF-2α and CYR61 in xenograft tumor tissues by Western blot and immunohistochemistry. As shown in **Figure 6D and Supplementary Figure S4D**, the xenograft tumor tissues derived from OS-RC-2-MIIP and 786-O-MIIP cells had lower levels of CYR61 and HIF-2α protein than those derived from the control cells. Nevertheless, silencing MIIP in 786-O cells led to an increase in CYR61 and HIF-2α protein levels (**Figure 6D**). The immunohistochemistry staining results showed similar alterations (**Figure 6C and Supplementary Figure S4C**). Together, these data indicated that MIIP acts as a novel tumor suppressor that inhibits ccRCC proliferation and angiogenesis, both *in vitro* and *in vivo*.

### MIIP is expressed at low levels in RCC and is associated with progression, prognosis, and the expression of CYR61 and HIF-2α

**Figure 7:**
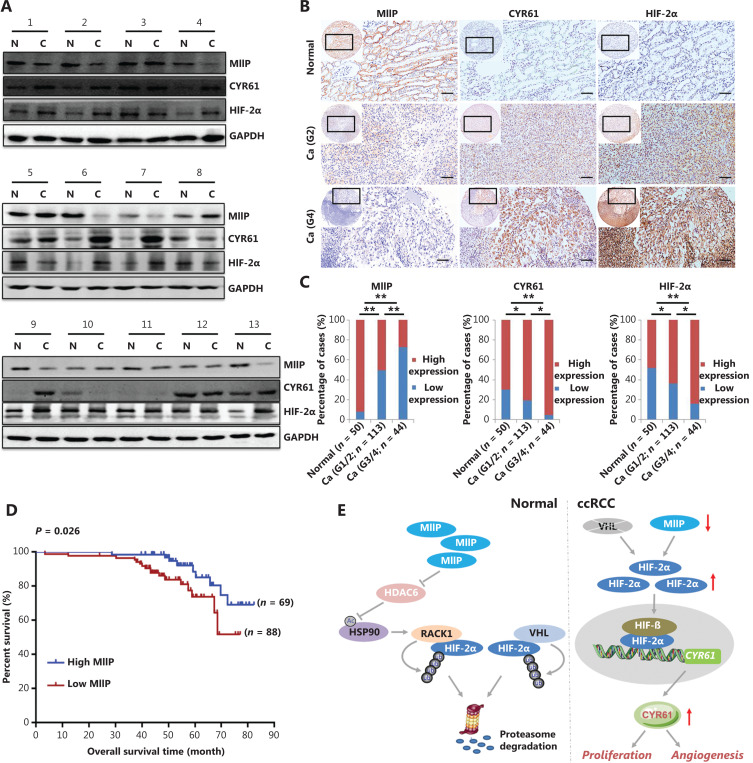
MIIP is weakly expressed in RCC and associated with progression, prognosis, and the expression of CYR61 and HIF-2α. (A) MIIP, CYR61, and HIF-2α expression in 13 pairs of RCC tissues *vs.* adjacent non-tumor tissues was detected by Western blot. C: RCC tissue; N: adjacent non-tumor tissue. (B) MIIP, CYR61, and HIF-2α expression in an RCC tissue microarray was detected with immunohistochemistry (scale bar: 50 μm). (C) MIIP, CYR61, and HIF-2α expression levels were quantified with a scoring system. The staining score was calculated by multiplying the stained area (%) score and the intensity score. Expression group: low, score < 9; high, score ≥ 9. Stage refers to WHO histological grade. *P < 0.05; **P < 0.01, *χ*^2^ test. (D) The Kaplan-Meier curve depicts the relationship between MIIP expression level and OS of patients with RCC, and the *P*-value was calculated with a stratified log rank test (*P* = 0.026). (E) Schematic representation of the MIIP/HIF-2α/CYR61 axis in ccRCC. In normal cells, MIIP promotes HSP90 acetylation through inhibiting HDAC6 activity, thus impairing HSP90’s chaperone function and its binding to HIF-2α, which in turn causes RACK1 binding and subsequent HIF-2α degradation. Meanwhile, under normoxia, VHL promotes HIF-2α ubiquitination and degradation in an oxygen-dependent manner. In RCC, VHL deficiency and MIIP downregulation together cause HIF-2α accumulation, thereby leading to overexpression of CYR61, which in turn contributes to RCC progression.

To evaluate the clinical relevance of MIIP in RCC, we collected 13 pairs of human RCC specimens together with their corresponding adjacent non-tumor tissues, and detected MIIP levels by Western blot. As shown in **Figure 7A**, 8 of 13 (61.54%) pairs of samples showed significantly lower MIIP protein levels in tumor tissues *vs.* adjacent normal tissues, whereas 4 of 13 (30.77%) showed no difference, and 1 of 13 (7.69%) showed greater MIIP levels. Moreover, among 8 pairs of samples with reduced MIIP, 7 pairs showed clearly upregulated HIF-2α and CYR61 expression in tumor tissues (**Figure 7A**). Interestingly, only 1 sample with MIIP upregulation displayed HIF-2α and CYR61 downregulation (**Figure 7A**).

We then analyzed MIIP expression in tissue microarray slides consisting of 157 RCC samples and 50 normal renal samples. On the basis of the staining scores of MIIP in tissues, the specimens were divided into a low MIIP group and a high MIIP group. We found that 92.00% (46/50) of normal renal tissues, but only 43.95% (69/157) of RCC tissues, exhibited high expression of MIIP (*P* < 0.01) (**Figure 7B–C**). These data further demonstrated that MIIP expression is downregulated in RCC *vs.* normal tissues.

Finally, the association of MIIP expression levels with clinical pathology, clinical outcomes of patients with RCC, and the expression status of HIF-2α and CYR61 was further analyzed. As shown in **Table 1**, although the MIIP expression levels were not correlated with age, gender, tumor size, or TNM stage (*P >* 0.05), low expression of MIIP was significantly associated with a higher histological grade (*P* < 0.01) (WHO histological grade, **Figure 7B–C**) and distant metastasis (*P* < 0.05) (**Table 1**). In addition, Kaplan-Meier analysis showed that higher MIIP expression in the tumor tissues from patients with ccRCC correlated with longer overall survival times (*P* = 0.026) (**Figure 7D**). Moreover, the HIF-2α and CYR61 expression was much higher in RCC tissues than in normal renal tissues (*P* < 0.05) (**Figure 7B–C**). Finally, the expression of HIF-2α and CYR61 was significantly but negatively associated with that of MIIP (*P* = 0.033; *P* = 0.024) (**Table 1**). Collectively, these findings further confirmed the dysregulation of the MIIP/HIF-2α/CYR61 pathway in ccRCC progression (**Figure 7E**).

**Table 1:** Correlation of MIIP expression to clinicopathological features and the expression of CYR61 and HIF-2α in RCC

Parameters	MIIP		*P* value
	High (≥ 9)	Low (< 9)	
Age (years)			
≤ 55	35 (42.68%)	47 (57.32%)	0.738
> 55	34 (45.33%)	41 (54.67%)	
Gender			
Male	45 (44.12%)	57 (55.88%)	0.954
Female	24 (43.64%)	31 (56.36%)	
Tumor size (cm)			
≤ 5	42 (48.84%)	44 (51.16%)	0.174
> 5	27 (38.03%)	44 (61.97%)	
TNM stage			
I and II	62 (46.27%)	72 (53.73%)	0.158
III and IV	7 (30.43%)	16 (69.57%)	
Histological grade			
G1 and G2	57 (50.44%)	56 (49.56%)	0.009
G3 and G4	12 (27.27%)	32 (72.73%)	
pr-Metastasis			
M0	66 (46.48%)	76 (53.52%)	0.049
M1	3 (20.00%)	12 (80.00%)	
CYR61 expression			
High (≥ 9)	54 (40.60%)	79 (59.40%)	0.046
Low (< 9)	15 (62.50%)	9 (37.50%)	
HIF-2α expression			
High (≥ 9)	42 (38.53%)	67 (61.47%)	0.039
Low (< 9)	27 (56.25%)	21 (43.75%)	

Statistical significance was calculated with the *χ*^2^ test. TNM stage refers to tumor node metastasis; histological grade refers to WHO histological grade; pr-Metastasis refers to postoperative recurrent-metastasis.

## Discussion

Emerging studies indicate that MIIP is downregulated in a variety of human tumors, including prostate cancer, glioma, lung cancer, colon cancer, endometrial carcinoma, and pancreatic cancer; moreover, its expression level is correlated with advanced clinical stage and patient prognosis^20–23^. Functionally, MIIP has been well documented to regulate cell migration, invasion, proliferation, cytoskeletal remodeling, and genome stability^16,17,19,24^. Herein, we report the first evidence that MIIP levels are lower in RCC samples than in normal renal tissues, and that its expression is associated with histological grade, metastasis, and prognosis. MIIP was found to inhibit the proliferation and proangiogenic activity of ccRCC cells. This study not only strengthens the evidence that MIIP acts as a tumor suppressor in different types of cancer but also provides the first evidence that MIIP is a negative regulator of angiogenesis.

Angiogenesis is a key feature of ccRCC, mainly because of its molecular hallmark of VHL inactivation, which causes HIF activation and consequent transcription of a variety of target genes. Among the HIF target genes, VEGF, PDGF, and FGF are well-known angiogenesis inducers^36^. Given the important role of angiogenesis in ccRCC, antiangiogenic drugs have been used as a major therapeutic approach for advanced ccRCC, mainly including antibodies against VEGF and tyrosine kinase inhibitors targeting VEGFR or other kinases^25,37^. Although such therapies have dramatically improved the prognosis of patients with RCC, primary or acquired resistance remains a major clinical problem^38,39^. Potential resistance mechanisms include HIF- or non-HIF-mediated alternative proangiogenic pathways and/or increased invasiveness^40^. To combat this resistance, emerging strategies include combination therapies and/or new agents such as HIF-2α inhibitors^38^. Here, we revealed the first evidence that MIIP functions as an endogenous HIF-2α antagonist and angiogenesis inhibitor by enhancing HIF-2α degradation and thus attenuating the HIF-2α-CYR61 axis.

Like its analog HIF-1α, HIF-2α undergoes proteasomal degradation in an oxygen/PHD/pVHL-dependent manner and consequently accumulates under hypoxia or pVHL inactivation^31^. In addition to this widely accepted mechanism, HIFα can be degraded in an oxygen/PHD/pVHL-independent manner. RACK1, an HIF-1/2α interacting protein, facilitates proteasomal degradation of HIF-1/2α through competition with HSP90 binding and recruitment of the Elongin-C/B ubiquitin ligase complex^32^. In contrast, HSP90, a molecular chaperone^41^, competes with RACK1 for binding the HIF PAS-A domain, thereby stabilizing HIF-1/2α^42–44^. Notably, the chaperone function of HSP90 can be inactivated by acetylation^33^. Here, we found that the MIIP-promoted HIF-2α degradation was dependent on RACK1 and proteasomal activity, because RACK1 siRNAs or proteasome inhibitors abrogated this degradation. Furthermore, we demonstrated that under MIIP overexpression, HSP90 acetylation was elevated, HSP90-HIF-2α binding was reduced, and RACK1-HIF-2α binding was increased. Given that HSP90 is an HDAC6 substrate, and MIIP inhibits HDAC6^15,19,45,46^, we concluded that increased acetylation of HSP90 results from MIIP-mediated inhibition of HDAC6. Interestingly, a recent study on pancreatic cancer has reported that MIIP leads to destabilization of HIF-1α by inhibiting the deacetylase activity of HDAC6 and thereby enhancing HIF-1α acetylation^46^. Whether MIIP regulates HIF-1α and HIF-2α through these 2 mechanisms simultaneously, differentially, or in a cancer-type dependent manner remains to be investigated.

Although HIF-1α and HIF-2α share significant homology, a tripartite structure, and a degradation pathway, HIF-2α, but not HIF-1α, has been clearly established as the dominant isoform that plays a crucial role in ccRCC initiation and progression^8,10,12,47,48^. Importantly, eliminating HIF-2α is sufficient to suppress VHL-defective tumor growth^49,50^. These findings highlight targeting HIF-2α as a promising potential strategy for ccRCC treatment. Indeed, small molecule HIF-2α antagonists have recently been developed and entered clinical trials^51,52^. However, acquired resistance has also been reported to emerge in sensitive ccRCC cells after prolonged treatment, owing to HIF-2α mutation^51^. Our finding that MIIP acts as an endogenous HIF-2α antagonist by promoting its destruction at the protein level strongly suggests that this type of resistance might be overcome after MIIP is harnessed for therapeutic purposes.

Most studies have indicated increased expression of CYR61 and its association with progression and poor prognosis in various cancers^53^, such as ovarian cancer^54^, prostate cancer^55^, and breast cancer^56^. CYR61 exerts its oncogenic effect by regulating cell proliferation, migration, invasion/metastasis, and vascularity^53–56^. Accordingly, we confirmed that CYR61 is upregulated in ccRCC, and promotes proliferation and angiogenesis. Importantly, we found that CYR61, a downstream target of HIF-2α, is a key mediator and contributor to the role of MIIP in ccRCC, because CYR61 abrogated the MIIP-induced effects on cellular behaviors. However, overexpression of MIIP in 786-O and OS-RC-2 cells unexpectedly had slight or no inhibitory effects on VEGF, a well-known target gene of HIFs and an important player in ccRCC. MIIP might potentially play a positive role in VEGF transcription or translation through an unknown mechanism.

## Conclusions

Our combined *in vitro* functional and molecular studies and *in vivo* xenograft mouse model and clinical sample analyses provide strong evidence that MIIP is a potential tumor suppressor in ccRCC. MIIP exerts its function by promoting HSP90 acetylation *via* HDAC6 inhibition, thus favoring competitive RACK1 binding to HIF-2α and subsequent HIF-2α degradation, which in turn attenuates downstream CYR61 signaling and its oncogenic effects. MIIP downregulation, together with VHL deficiency, contributes to or exacerbates the accumulation of HIF-2α in ccRCC, thereby causing aberrant expression of CYR61 and ccRCC progression (**Figure 7E**). Our data reveal a novel mechanism of MIIP in ccRCC and suggest that modulation of MIIP may serve as a new therapeutic strategy for ccRCC treatment.
