# Supplementary material for: MIIP inhibits clear cell renal cell carcinoma proliferation and angiogenesis via negative modulation of the HIF-2α-CYR61 axis
Source: Cancer Biol Med. 2021 Dec 22;19(6):818–35. doi: 10.20892/j.issn.2095-3941.2020.0296 (PMC9257321; doi:10.20892/j.issn.2095-3941.2020.0296)
Supplement: Supplementary file 1 [file cbm-19-818-s001.pdf]

Supplementary materials

Supplementary methods

Cell culture

The human ccRCC cell lines 769-P, ACHN, Caki-1, and Caki-2 were purchased from the Chinese Academy of Sciences Cell Bank. The 769-P and ACHN cells were maintained in RPMI-1640 medium (Invitrogen, USA), and the Caki-1 and Caki-2 cells were maintained in McCoy's 5A medium (Invitrogen, USA). All media were supplemented with 10% fetal bovine serum (Gibco, USA), penicillin, and streptomycin, and incubated in a 37 °C humidified incubator with 5% CO_2. All cell lines were authenticated, and tested bacteria and mycoplasma free during the past 3 months.

Vector construction and lentivirus packaging

The lentiviral pLEX-MIIP-HA vector was constructed by amplification of the CDS fragment of MIIP with PCR with the primers listed below and cloning into the pLEX-HA-MCS vector. For lentiviral packaging, pLEX-MIIP-HA/pLEX-HA (1 μg) was transfected into HEK-293T cells together with the packaging system (0.2 μg VSVG and 1 μg Δ8.9) with LipofectamineTM^{2000} (Thermo Scientific, Waltham, MA) according to the manufacturer's instructions. pLKO.1-shMIIP were generated by annealing the MIIP-targeting shRNA oligonucleotides as listed below and cloning them into the pLKO.1 vector. The lentiviral particles were obtained by co-transfection of HEK-293T cells with pLKO.1-shMIIP (or pLKO.1-Scramble shRNA) (1 μg) and the packaging system (0.75 μg psPAX2 and 0.25 μg pMD2.G).

Collection of secreted proteins

A total of 2 × 10^5 HK-2 or ccRCC cells were seeded in 10 cm dishes and grown to 70% confluency in RPMI-1640 containing 10% serum, then cultured for another 48 h with RPMI-1640 medium containing 1% serum. The cell culture supernatants were harvested and centrifuged at 1,000 r/min for 5 min to remove cell debris (4 °C). Then 15 mL supernatant was added to Amicon Ultra-15 Centrifugal Filter Unit tubes (30K, Merck Millipore, USA) and centrifuged at 14,000 g for 10 min (4 °C). The liquid remaining in the upper layer after centrifugation contained the proteins secreted in the medium. Then 2 μL of the secreted protein was collected for protein quantification, and the rest was used for Western blot.

Table S1 Primers used for plasmid construction

Plasmid name	Primers (5′–3′)
pLEX-MIIP-HA	Forward: CGGACTAGT ATGGTGGAGGCTGAGGAAC
	Reverse: CCGCTCGAG TCCTCCCTCTCTGGAACTCA
pLKO.1-shMIIP 1#	Forward: CCGGGTGTACTGTTACCGTGTCAACCTCGAGGTTGACACGGTAACAGTACACTTTTTG
	Reverse: AATTCAAAAAGTGTACTGTTACCGTGTCAACCTCGAGGTTGACACGGTAACAGTACAC
pLKO.1-shMIIP 2#	Forward: CCGGCGTGGAGGAAGACCATGAATGCTCGAGCATTCATGGTCTTCCTCCACGTTTTTG
	Reverse: AATTCAAAAACGTGGAGGAAGACCATGAATGCTCGAGCATTCATGGTCTTCCTCCACG

Table S2 The siRNA used in the experiments

siRNA name	Sequences (5′–3′)
si-NC	Sense: UUCUCCGAACGUGUCACGUTT
	Antisense: ACGUGACACGUUCGGAGAATT
si-CYR61 1#	Sense: GGCAGACCCUGUGAAUAUATT
	Antisense: UAUAUUCACAGGGUCUGCCTT
si-CYR61 2#	Sense: CCAGAAAUGUAUUGUUCAATT
	Antisense: UUGAACAAUACAUUUCUGGTT
si-HIF-2α 1#	Sense: CUCCUCAGUUUGCUCUGAATT
	Antisense: UUCAGAGCAAACUGAGGAGTT
si-HIF-2α 2#	Sense: CAGAACUGAUUGGUUACCATT
	Antisense: UGGUAACCAAUCAGUUCUGTT
si-RACK11#	Sense: CUGGGUGUGUGCAAAUACATT
	Antisense: UGUAUUUGCACACACCCAGTT
si-RACK1 2#	Sense: CCCACUUUGUUAGUGAUGUTT
	Antisense: ACAUCACUAACAAAGUGGGTT

Table S3 Primers used for qRT-PCR analysis

Gene name	Primers (5′–3′)
MIIP	Forward: ATACCTGGGCTATGACTGGATT
	Reverse: AGTACACGCATTCATGGTCTTC
CYR61	Forward: CTCGCCTTAGTCGTCACCC
	Reverse: CGCCGAAGTTGCATTCCAG
HIF2A	Forward: CGGAGGTGTTCTATGAGCTGG
	Reverse: AGCTTGTGTGTTCGCAGGAA
GAPDH	Forward: CCATCTTCCAGGAGCGAGATC
	Reverse: GCCTTCTCCATGGTGGTGAA

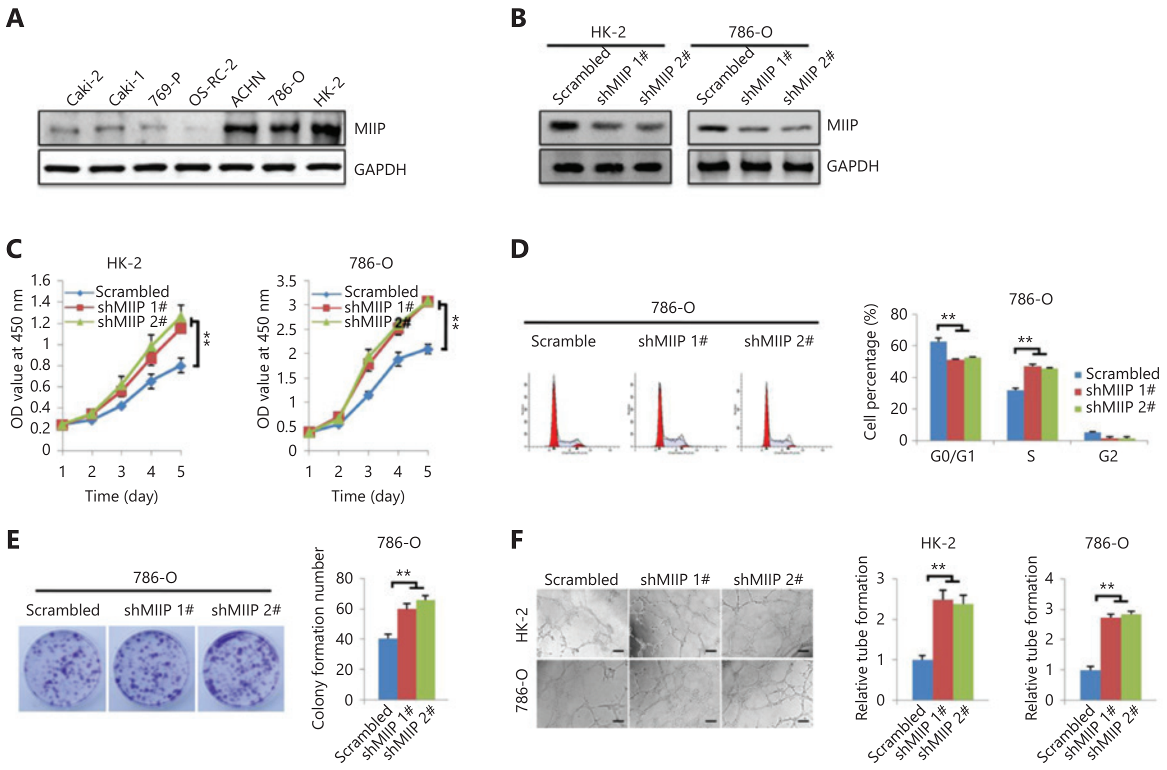

Figure S1 MIIP knockdown promotes the malignant attributes of ccRCC cells *in vitro*. (A) MIIP expression in HK-2 and ccRCC cell lines was detected by Western blot. (B–F) Cells with stable MIIP knockdown (HK-2-shMIIP 1#, HK-2-shMIIP 2#; 786-O-shMIIP 1#, 786-O-shMIIP 2#) and control (HK-2-Scramble and 786-O-Scramble) cells were generated by lentiviral infection. (B) MIIP expression was detected with Western blot. (C) Viability was measured with CCK-8. (D) Cell cycle profiles were analyzed by flow cytometry. (E) Colony formation ability was detected with colony formation assays. (F) Proangiogenic activity was measured with HUVEC tube formation assays, and the HUVECs were cocultured with conditioned medium for 5 h (scale bar: 100 μm). The data are represented as mean ± SD, $n = 3$. *$P < 0.01$, **$P < 0.01$.

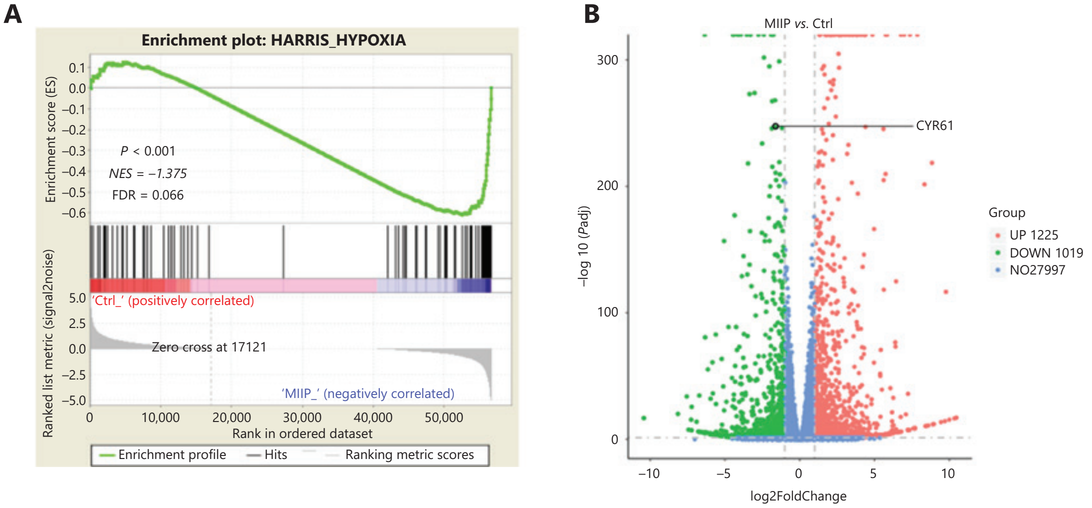

Figure S2 MIIP negatively regulates hypoxia gene sets and CYR61 expression. (A) RNA-seq analyses were performed in triplicate on 786-O-MIIP and 786-O-Vector cells, and the datasets were analyzed by GSEA. A portion of the hypoxia pathway GSEA (hallmark gene sets) of the RNA-seq dataset is shown. (B) Volcano plots of RNA-seq and the *CYR61* expression.

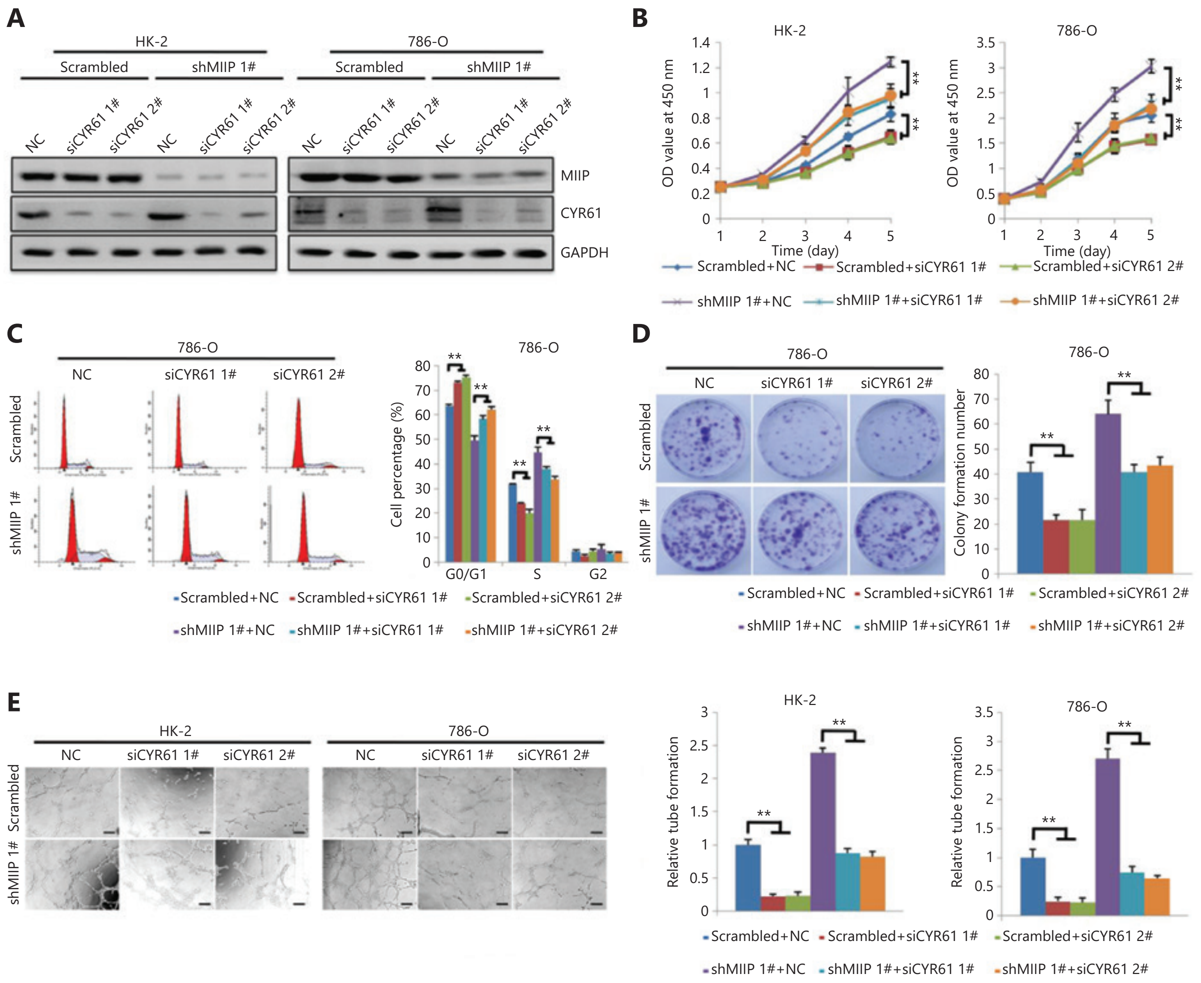

Figure S3 Knockdown of CYR61 reverses the effects of MIIP knockdown on cellular behaviors. Cells with stable MIIP knockdown and control cells were transfected with siRNA targeting CYR61 (siCYR61 1# or siCYR61 2#) or control (siNC), respectively. (A) Protein levels of MIIP and CYR61 were detected by Western blot. (B) Viability was measured with CCK-8. (C) Cell cycle profiles were analyzed by flow cytometry. (D) Colony formation ability was detected with colony formation assays. (E) Proangiogenic activity was measured with HUEVC cell tube formation assays, and the HUVECs were cocultured with conditioned medium for 5 h (scale bar: 100 μm). The data are represented as mean ± SD, $n = 3$. $^{**}P < 0.01$.

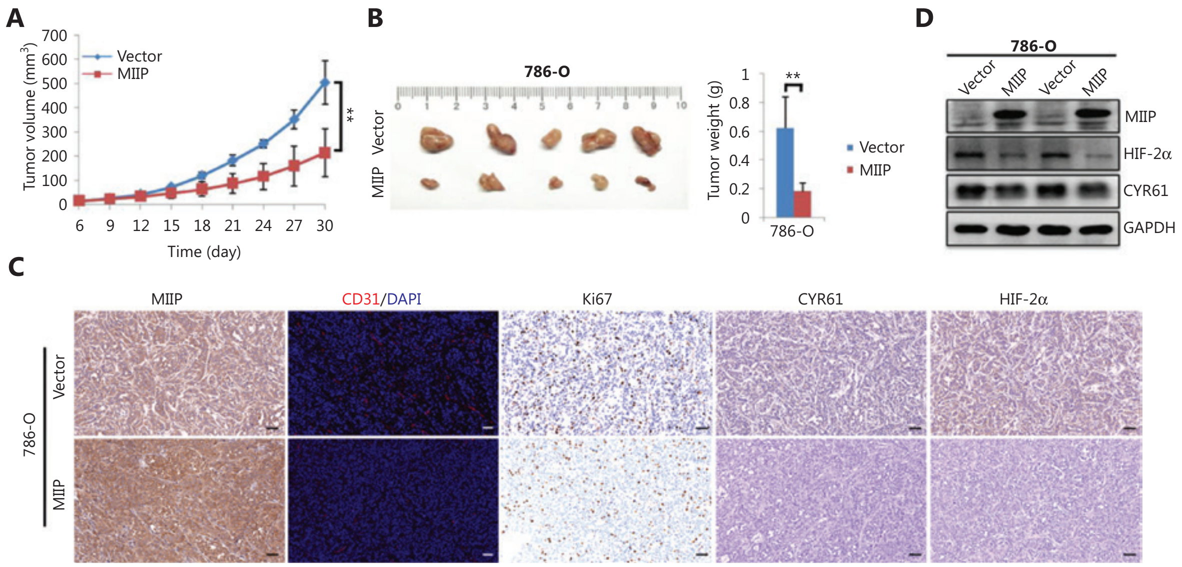

Figure S4 MIIP suppresses tumor growth and angiogenesis *in vivo*. 786-O-MIIP and 786-O-Vector cells were bilaterally subcutaneously injected into the groin in nude mice (n = 5/group). (A) Tumor growth was monitored by calculation of tumor volumes with the formula width 2 × length × 0.5 along time. (B) The tumor xenografts were photographed, and their weights were measured at the end of the experiment. (C) MIIP, CD31, Ki67, HIF-2α, and CYR61 levels in xenograft tissues were examined by immunohistochemistry or immunofluorescence staining (scale bar: 50 μm). (D) MIIP, HIF-2α, and CYR61 levels in xenograft tissues were detected by Western blot. In (A, B), the data are represented as mean ± SD, n = 5. $^{**}P < 0.01$.
